# Differential effect of meteorological factors and particulate matter with ≤ 10-µm diameter on epistaxis in younger and older children

**DOI:** 10.1038/s41598-022-25630-3

**Published:** 2022-12-05

**Authors:** Il-Youp Kwak, Kyung Soo Kim, Hyun Jin Min

**Affiliations:** 1 Department of Applied Statistics, Chung-Ang University, 224-1 Heukseok-Dong, Dongjak-Gu, Seoul, 156-755 South Korea; 2 Department of Otorhinolaryngology-Head and Neck Surgery, Chung-Ang University College of Medicine, 224-1 Heukseok-Dong, Dongjak-Gu, Seoul, 06973 South Korea

**Keywords:** Environmental sciences, Diseases, Risk factors

## Abstract

The differential effect of meteorological factors and air pollutants on pediatric epistaxis in younger and older children has not been evaluated. We evaluated the distribution of pediatric epistaxis cases between younger (0–5 years) and older children (6–18 years). Subsequently, we assessed and compared the effects of meteorological variables and the concentration of particulate matter measuring ≤ 10 μm in diameter (PM10) on hospital epistaxis presentation in younger and older children. This retrospective study included pediatric patients (n = 326) who presented with spontaneous epistaxis between January 2015 and August 2019. Meteorological conditions and PM10 concentration were the exposure variables, and data were obtained from Korea Meteorological Administration 75. The presence and cumulative number of epistaxis presentations per day were considered outcome variables. Air temperature, wind speed, sunshine duration, and PM10 concentration in younger children, and sunshine duration and air pressure in older children, significantly correlated with the presence of and cumulative number of epistaxis presentations per day. The PM10 concentration was not a significant factor in older children. Thus, meteorological factors and PM10 concentration may differentially affect epistaxis in younger (0–5-year-olds) and older (6–18-year-olds) children. Risk factors for pediatric epistaxis should be considered according to age.

## Introduction

Particulate matter (PM) is a complex mixture of organic and inorganic compounds found in the atmosphere, in the gas, liquid, and solid phases^1^. Owing to the genotoxic, mutagenic, and carcinogenic effects of PM, exposure to PM is a risk factor for various diseases such as cardiovascular disease, stroke, and airway diseases^2^. The size of PM determines the target organ that it penetrates. PM10 (≤ 10 μm, coarse particles) is known to settle in the upper airway tract and trigger allergenic and irritating responses. In South Korea, the PM10 concentration is higher than that in North America, Western Europe, and Japan, and reports of a spontaneous increase in PM10 concentration are of major concern^3^.

Epistaxis is a common symptom in children, and it is reported that up to 60% of children will have had at least one epistaxis event by 10 years of age^4^. Various conditions associated with pediatric epistaxis have been evaluated. In terms of individual patient factors, age and allergic rhinitis have been shown to be significantly associated with hospital epistaxis presentation in children^5^. Regarding environmental conditions, several meteorological factors have been suggested to be important in pediatric epistaxis. For example, temperature and air visibility have been reported as vital climate variables in pediatric epistaxis^6^. Humidity has likewise been linked to pediatric epistaxis^7^. In addition to meteorological factors, air pollutants such as PM10 have also been recently shown to be associated with the incidence of epistaxis in children^4,8^.

The daily lives of preschool children (0–5 years of age) differ from that of school-aged children (6–18 years) in this country. The life of younger children might be more dependent on the daily weather. Younger children might spend a considerable amount of time outdoors during days with fair weather but might spend more time indoors during days with bad weather. Furthermore, the daily life of school-aged children tends to be more constant, with them spending a considerable amount of time indoors regardless of the weather.

### Objectives

We hypothesized that epistaxis, which is closely associated with meteorological factors and PM10, would show dissimilar patterns between younger and older children. This study aimed to evaluate the distribution of pediatric epistaxis cases among younger children aged 0–5 years and older children aged 6–18 years.

## Methods

### Study design

In this retrospective, single-center study, medical records of pediatric patients (age ≤ 18 years) who presented with spontaneous epistaxis at Chung-Ang University Hospital between January 2015 and August 2019 were reviewed. Patients who presented with secondary epistaxis due to bleeding disorders or a foreign body lodged in the nose were excluded from this study.

### Exposure variables

Meteorological conditions and PM10 concentration were the exposure variables, and the data were obtained from Korea Meteorological Administration 75 (https://web.kma.go.kr/eng/index.jsp). Data on air pressure (hPa), wind speed (m/s), air temperature (°C), relative humidity (%), sunshine duration (h), amount of solar radiation (MJ/m^2^), number of clouds (cloud cover) (1/10), and PM10 concentration (μg/m^3^) are daily reported, and we collected these daily reported data from January 1, 2015, to August 31, 2019, were analyzed^8^.

### Outcome variables

The presence of hospital epistaxis presentation and the cumulative number of epistaxis presentations per day were considered outcome variables.

### Statistical analyses

Exposure variables are presented as means with a 95% confidence interval. Univariate and multivariate logistic regression analyses were performed to identify significant explanatory variables associated with hospital epistaxis presentation. Univariate and multivariate Poisson regression analyses were performed to identify significant explanatory variables associated with the cumulative number of hospital epistaxis presentations per day. The correlation was assessed using Pearson’s correlation analysis. A two-tailed *p* value of < 0.05 was considered to be indicative of statistical significance. Statistical analyses were performed using R version 3.6.2.

### Ethics statement

This study was approved by the Institutional Review Board of Chung-Ang University College of Medicine (2002-013-19303), and the requirement for informed consent was waived.

### Reporting guideline

This manuscript has been prepared in accordance with the STROBE guidelines for reporting observational studies.

## Results

### Summary characteristics of exposure and study population data

**Figure 1:**
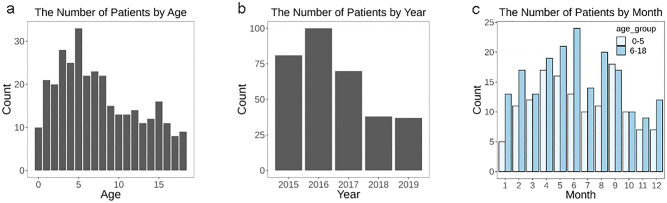
Age distribution of hospital epistaxis presentation from 2015 to 2019. (**a**) Age distribution of hospital epistaxis presentation. (**b**) Yearly distribution of hospital epistaxis presentation from 2015 to 2019. (**c**) Monthly distribution of hospital epistaxis presentation from 2015 to 2019.

A total of 326 children were enrolled in this study. The mean age was 7.62 ± 4.92 years. The number of children in the 0–5- and 6–18-year age groups was 137 and 189, respectively (Table 1). The number of hospital epistaxis presentations per day showed a decreasing pattern with age, reaching a peak with 33 visitors at the age of 5 years and then gradually decreasing to 9 visitors at the age of 18 years (Fig. 1a). When the number of epistaxis cases was reviewed yearly, we found that the number of annual visits by patients with epistaxis was 81 in 2015, 100 in 2016, 70 in 2017, 38 in 2018, and 37 in 2019 (Fig. 1b). We divided pediatric patients into two groups aged 0–5 and 6–18 years and found a few more patients in the 6–18-year age group during summer; however, the difference was not statistically significant (Fig. 1c).

**Table 1:** Characteristics of the study participants.

	N
No. of patients	326
Ratio of participants in the 0–5-year and 6–18-year age groups	137:189
**Age (years)**	
**All participants**	
Mean	7.62 (4.92)
Range	0.08–18
**0–5-year age group**	
Mean	3.04 (1.53)
Range	0.08–5
**6–18-year age group**	
Mean	10.95 (3.73)
Range	6–18

Supplementary Table S1 compares the monthly averages and 95% confidence intervals of explanatory variables (air temperature, wind speed, air pressure, humidity, sunshine duration, solar radiation amount, number of clouds, and PM10). All variables had seasonal fluctuations.

### Factors associated with the presence of daily hospital epistaxis presentation according to age groups

The associations between meteorological factors, including PM10 concentration and daily hospital epistaxis presentation, are presented in Table 2. In the 0–5-year age group, minimum temperature, mean wind speed, sunshine duration, and PM10 concentration were positively associated with the presence of hospital epistaxis presentation per day. In the 6–18-year age group, sunshine duration was positively associated with the presence of hospital epistaxis presentation, whereas the mean air pressure was negatively associated. Additionally, PM10 concentration was not a significant factor in the 6–18-year age group.

**Table 2:** Multivariate logistic regression analyses of meteorological factors, including PM10 concentration and the number of hospital epistaxis presentations per day between the 0–5- and 6–18-year age groups.

Parameter	Univariate analysis		Multivariate analysis	
	Odds ratio (95% CI)	*p* value	Odds ratio (95% CI)	*p* value
**0–5 years**				
Mean temperature (°C)	1.021 (1.003–1.039)	0.020		
Minimum temperature (°C)	1.016 (0.999–1.036)	0.071	1.026 (1.004–1.048)	0.022
Maximum temperature (°C)	1.025 (1.007–1.043)	0.007		
Temperature difference (°C)	1.124 (1.054–1.199)	< 0.001		
Mean air pressure (hPa)	0.986 (0.977–1.003)	0.227		
Maximum wind speed (m/s)	0.992 (0.979–1.005)	0.838		
Mean wind speed (m/s)	1.011 (0.914–1.117)	0.022	1.346 (1.022–1.771)	0.034
Minimum relative humidity (%)	1.298 (1.039–1.622)	0.137		
Mean relative humidity (%)	0.990 (0.977–1.003)	0.204		
Sunshine duration (h)	1.101 (1.043–1.161)	< 0.001	1.065 (1.005–1.130)	0.034
Solar radiation amount (MJ/m^2^)	1.042 (1.014–1.071)	0.003		
Number of clouds (1/10)	0.914 (0.858–0.973)	0.005		
PM10 (μg/m^3^)	1.005 (0.998–1.013)	0.127	1.009 (1.000–1.017)	0.040
**6–18 years**				
Mean temperature (°C)	1.014 (0.999–1.030)	0.066		
Minimum temperature (°C)	1.012 (0.997–1.027)	0.114		
Maximum temperature (°C)	1.016 (1.000–1.031)	0.043		
Temperature difference (°C)	1.051 (0.994–1.111)	0.079		
Mean air pressure (hPa)	0.978 (0.0958–0.998)	0.035	0.973 (0.950–0.997)	0.027
Maximum wind speed (m/s)	1.049 (0.964–1.142)	0.269		
Mean wind speed (m/s)	1.140 (0.929–1.398)	0.209		
Minimum relative humidity (%)	0.994 (0.983–1.006)	0.339		
Mean relative humidity (%)	0.996 (0.984–1.007)	0.463		
Sunshine duration (h)	1.053 (1.007–1.057)	0.021	1.078 (1.026–1.132)	0.003
Solar radiation amount (MJ/m^2^)	1.032 (1.007–1.057)	0.010		
Number of clouds (1/10)	0.975 (0.924–1.029)	0.359		
PM10 (μg/m^3^)	1.001 (0.994–1.110)	0.812		

CI: confidence interval, PM10: particulate matter measuring ≤ 10 μm in diameter.

### Factors associated with the cumulative number of hospital epistaxis presentations per day according to age groups

Next, we evaluated the effects of meteorological factors, including PM10 concentration, on the cumulative number of hospital epistaxis presentations per day (Table 3). In the 0–5-year age group, maximum air temperature, mean wind speed, sunshine duration, and PM10 concentration were positively associated with the daily cumulative number of epistaxis presentations. In the 6–18-year age group, sunshine duration was positively associated with the daily cumulative number of epistaxis presentations, whereas the mean air pressure was negatively associated. However, PM10 concentration was not significantly associated with the daily cumulative number of epistaxis presentations in the 6–18-year age group.

**Table 3:** Univariate and multivariate Poisson regression analyses of meteorological factors, including PM10 concentration and the cumulative number of hospital epistaxis presentations per day between the 0–5- and 6–18-year age groups.

Parameter	Univariate analysis		Multivariate analysis	
	Odds ratio (95% CI)	*p* value	Odds ratio (95% CI)	*p* value
**0–5 years**				
Mean temperature (°C)	1.021 (1.004–1.038)	0.016		
Minimum temperature (°C)	1.016 (1.000–1.032)	0.057		
Maximum temperature (°C)	1.024 (1.007–1.041)	0.005	1.028 (1.009–1.048)	0.003
Temperature difference (°C)	1.112 (1.047–1.181)	< 0.001		
Mean air pressure (hPa)	0.984 (0.962–1.006)	0.156		
Maximum wind speed (m/s)	1.019 (0.928–1.119)	0.690		
Mean wind speed (m/s)	1.259 (1.024–1.547)	0.029	1.312 (1.052–1.635)	0.016
Minimum relative humidity (%)	0.992 (0.979–1.004)	0.183		
Mean relative humidity (%)	0.994 (0.981–1.006)	0.299		
Sunshine duration (h)	1.092 (1.038–1.148)	< 0.001	1.063 (1.010–1.118)	0.019
Solar radiation amount (MJ/m^2^)	1.039 (1.013–1.066)	0.003		
Number of clouds (1/10)	0.924 (0.871–0.980)	0.008		
PM10 (μg/m^3^)	1.006 (0.999–1.013)	0.080	1.008 (1.002–1.015)	0.016
6–18 years				
Mean temperature (°C)	1.014 (1.004–1.028)	0.044		
Minimum temperature (°C)	1.013 (0.999–1.026)	0.070		
Maximum temperature (°C)	1.015 (1.002–1.029)	0.027		
Temperature difference (°C)	1.042 (0.991–1.095)	0.111		
Mean air pressure (hPa)	0.978 (0.959–0.996)	0.017	0.973 (0.954–0.992)	0.006
Maximum wind speed (m/s)	1.053 (0.976–1.136)	0.181		
Mean wind speed (m/s)	1.157 (0.966–1.386)	0.113		
Minimum relative humidity (%)	0.995 (0.985–1.006)	0.378		
Mean relative humidity (%)	0.995 (0.985–1.005)	0.334		
Sunshine duration (h)	1.047 (1.006–1.089)	0.024	1.047 (1.007–1.088)	0.021
Solar radiation amount (MJ/m^2^)	1.030 (1.009–1.053)	0.006		
Number of clouds (1/10)	0.977 (0.931–1.026)	0.355		
PM10 (μg/m^3^)	1.004 (0.998–1.010)	0.215	1.008 (1.000–1.012)	0.065

CI: confidence interval, PM10: particulate matter measuring ≤ 10 μm in diameter.

### Time-lag effect between hospital epistaxis presentation per day and meteorological factors

Finally, we analyzed the number of hospital epistaxis presentations per day and meteorological factors, including PM10 concentration, as measured during the previous 7 days to evaluate the time-lag effect (Table 4). In the 0–5-year age group, the presence of epistaxis was positively associated with air temperature on the day of the medical visit and during the previous 7 days (*p* < 0.05). The maximum value of correlation was 0.12 between the 5-day lagged epistaxis presentation and mean temperature. The presence of epistaxis was negatively associated with air pressure. The maximum air pressure was -0.08 for the 7-day lagged epistaxis presentation (*p* < 0.05) in the 0–5-year age group. In the 6–18-year age group, the presence of epistaxis was positively associated with temperature and solar radiation. The mean air temperature showed the highest correlation at 0.1 for the 1-day lagged epistaxis presentation (*p* < 0.05). Solar radiation also had the highest correlation of 0.1 with the 0-day lagged epistaxis presentation (*p* < 0.05). The presence of epistaxis was negatively associated with air pressure, and the maximum amount was −0.11 (Pearson’s correlation coefficient) at the 1-day lagged epistaxis presentation (*p* < 0.05) in the 6–18-year age group. PM10 concentration did not show any significant time-lag effect in either group.

**Table 4:** Time-lag effect between the number of epistaxis presentations per day and other variables.

	Mean temperature	Minimum temperature	Maximum temperature	Temperature difference	Mean air pressure	Maximum wind speed	Mean wind speed	Humidity	Minimum humidity	Sunshine duration	Solar radiation	Number of clouds	PM10
**0–5 years**													
Epistaxis count lag0	0.08*	0.08**	0.08**	0.02	− 0.07**	− 0.04	0.04	0.04	0.04	− 0.04	0	0.04	0.04
Epistaxis count lag1	0.08**	0.07**	0.09**	0.05	− 0.05	0.02	0.08**	0.02	0	0.02	0.06*	0	− 0.02
Epistaxis count lag2	0.11**	0.1**	0.11**	0.05	− 0.06*	− 0.01	0.01	0.04	0.03	0.02	0.05	− 0.01	0.01
Epistaxis count lag3	0.11**	0.11**	0.11**	0.01	− 0.07**	− 0.05	− 0.01	0.07**	0.07**	0.01	0.02	0.01	0
Epistaxis count lag4	0.1**	0.09**	0.09**	0.02	− 0.07**	− 0.06	0.01	0.02	0.03	0	0.03	0.03	− 0.04
Epistaxis count lag5	0.12**	0.11**	0.12**	0.02	− 0.1**	− 0.03	0.02	0.04	0.02	− 0.04	0.02	0.08**	− 0.01
Epistaxis count lag6	0.1**	0.1**	0.09**	− 0.02	− 0.07**	− 0.06*	− 0.02	0.09**	0.07**	− 0.05	− 0.02	0.07*	0.06*
Epistaxis count lag7	0.08**	0.08**	0.07**	− 0.02	− 0.08**	− 0.05	0.02	0.07**	0.09**	− 0.04	− 0.03	0.08**	0
**6–18 years**													
Epistaxis count lag0	0.1**	0.09**	0.1**	0.04	− 0.1**	0.02	0.03	− 0.01	− 0.01	0.04	0.1**	0.01	− 0.02
Epistaxis count lag1	0.1**	0.1**	0.09**	0	− 0.11**	0.01	0.02	0.02	0	− 0.03	0.05	0.06	− 0.02
Epistaxis count lag2	0.08**	0.07**	0.09**	0.06	− 0.08**	− 0.01	0.03	0.02	− 0.01	0.04	0.08**	− 0.02	0.02
Epistaxis count lag3	0.07**	0.07*	0.07*	0.01	− 0.05	0	0.02	− 0.01	0.02	0.03	0.07**	− 0.01	− 0.05
Epistaxis count lag4	0.06	0.06	0.05	0	− 0.05	0.03	0.01	0.02	0	0	0.03	0.01	− 0.05
Epistaxis count lag5	0.05	0.06*	0.05	− 0.03	− 0.05	0.03	0.03	0.04	0.03	0.05	0.06*	0	− 0.03
Epistaxis count lag6	0.06*	0.06*	0.07*	0.03	− 0.08**	0.03	0.03	− 0.03	− 0.04	0.04	0.07**	0.01	− 0.02
Epistaxis count lag7	0.07**	0.06*	0.08**	0.07**	− 0.07**	0.04	− 0.01	0.01	− 0.02	0.08**	0.1**	− 0.05	− 0.03

*p value < 0.1, **p value < 0.05.

## Discussion

Our study evaluated the differential effects of meteorological factors and PM10 concentration on hospital epistaxis presentation in younger and older children. To the best of our knowledge, this is the first study to compare the differential effects of meteorological factors, including PM10, on epistaxis between younger and older children.

Among the studied meteorological factors, air temperature and wind speed were significant factors for epistaxis in younger children, while air pressure was an important factor for older children. Sunshine duration was positively associated with hospital epistaxis presentation in both groups. Regarding the differential effects of meteorological factors, we believe that as younger children spend more time outdoors than older children who regularly go to school, they would be more severely affected by the daily weather. Therefore, it would be natural to expect that temperature, rain, and wind would be more strongly associated with epistaxis in younger children than older children. However, contrary to our expectations, humidity was not a significant factor for younger or older children in our study. Although the reported effect of humidity on epistaxis was not consistent in previous studies, several studies have indicated humidity to be an important factor in epistaxis^6,9,10^. Therefore, the role of humidity needs to be further evaluated in future studies based on a larger population.

PM10 is associated with many adverse health effects, such as increased mortality, hospitalization, emergency care, and outpatient rate, owing to the damage to the respiratory system^11,12^. Children are a susceptible population, and existing studies have found increased adverse effects on children’s respiratory systems^13^. Although meteorological factors have been considered important in the pathogenesis of epistaxis, PM10 has not been extensively evaluated, especially in children. Several mechanisms may explain why the increase in PM10 concentration is associated with the risk of pediatric epistaxis. Components of PM10 could stimulate the upper airway mucosa, leading to inflammatory responses such as cytokine release^14^. The nasal mucosa of children persistently exposed to PM10 exhibited basal cell hyperplasia, reduced number of ciliated and goblet cells, and neutrophilic epithelial infiltration, which could result in epistaxis^15^.

This study was conducted in South Korea, which has a relatively higher concentration of PM10 than other countries, and PM10 concentration is seen as an important social issue. In addition, we enrolled subjects with epistaxis before the emergence of the coronavirus disease to exclude the effect of wearing masks on the results. Our findings suggest that the effects of PM10 on children not wearing masks are different between younger and older children.

When compared with other meteorological factors, PM10 concentration did not show any time-lag effect in younger or older children. Temperature and air pressure showed a time-lag effect during the previous 7 days in the 0–5-year age group. Temperature, solar radiation, and air pressure showed a time-lag effect during the previous 0–1 day in the 6–18-year age group. However, PM10 did not show a significant time-lag effect in either group. A previous study found some correlation between air pollutants and the incidence of pediatric epistaxis; however, it did not show any obvious delayed effect^4^. No conclusive data have yet been published on the time-lag effect of PM10 concentration, and further study is warranted.

Our study has some limitations. First, the survey was based in a single institute, which is a tertiary referral center, and the generalizability of our findings might be limited. A large population-based multicenter study is needed in the future to confirm our findings. Second, this is a retrospective study, and we could not collect further specific information that might be associated with pediatric epistaxis. The presence of allergic rhinitis or acute respiratory infections and the effects of confounders such as traffic, which may lead patients to go to medical centers other than the hospital, were not evaluated.

In conclusion, the effect of meteorological factors and PM10 concentration on epistaxis was different between younger (0–5 years) and older (6–18 years) children. Our results suggests that environmental factors could differentially affect younger and older children. More attention must be paid to PM10 concentration in younger children, who are more vulnerable to adverse health effects than older children, to reduce cases of hospital epistaxis presentation.

## Supplementary Information

Supplementary Information.

## Data Availability

The datasets used and analyzed during the current study are available from the corresponding author on reasonable request.
