# Supplementary material for: Differential effect of meteorological factors and particulate matter with ≤ 10-µm diameter on epistaxis in younger and older children
Source: Sci Rep. 2022 Dec 5;12:21029. doi: 10.1038/s41598-022-25630-3 (PMC9723103; doi:10.1038/s41598-022-25630-3)
Supplement: Supplementary file 1 — Supplementary Information. [file 41598_2022_25630_MOESM1_ESM.docx]

Supplementary Table S1. Comparison of the monthly average of explanatory variables (temperature, wind speed, air pressure, humidity, sunshine duration, solar radiation amount, number of clouds, and PM10)

| **Month** | **Mean temperature (°C)** | **Minimum temperature (°C)** | **Maximum temperature (°C)** | **Temperature difference (°C)** | **Mean air pressure (hPa)** | **Maximum wind speed (m/s)** | **Mean wind speed (m/s)** | **Minimum relative humidity (%)** | **Mean relative humidity (%)** | **Sunshine duration (h)** | **Solar radiation amount (MJ/m^2^)** | **Number of clouds (1/10)** | **PM10 (μg/m^3^)** |
| --- | --- | --- | --- | --- | --- | --- | --- | --- | --- | --- | --- | --- | --- |
| 1 | -2.09±4.36 | -5.90±4.53 | 2.46±4.37 | 8.37±1.97 | 1014.30±4.45 | 5.35±1.89 | 2.22±0.86 | 32.10±11.56 | 51.60±12.42 | 6.53±3.02 | 7.79±2.63 | 3.74±2.87 | 51.31±23.27 |
| 2 | 0.04±3.95 | -4.21±3.98 | 5.04±4.23 | 9.26±2.74 | 1012.85±5.00 | 5.72±1.66 | 2.39±0.84 | 29.00±12.06 | 51.09±14.50 | 6.94±3.50 | 10.71±4.13 | 3.90±3.07 | 49.79±19.88 |
| 3 | 6.91±3.96 | 2.25±4.02 | 12.62±4.57 | 10.37±2.81 | 1009.37±4.42 | 6.06±2.12 | 2.40±0.81 | 26.65±11.51 | 50.41±12.93 | 7.71±3.63 | 14.18±4.55 | 4.00±2.98 | 59.17±30.76 |
| 4 | 13.34±3.36 | 8.30±3.19 | 19.21±4.28 | 10.91±3.43 | 1004.43±4.75 | 6.31±1.96 | 2.42±0.77 | 28.23±15.33 | 52.31±15.88 | 7.89±4.35 | 16.71±6.72 | 4.58±3.15 | 50.13±23.48 |
| 5 | 19.23±2.74 | 13.90±2.59 | 25.21±3.65 | 11.31±3.13 | 1001.07±4.75 | 6.13±2.11 | 2.35±0.80 | 30.45±14.96 | 53.28±15.11 | 9.38±4.25 | 20.40±6.91 | 4.29±3.16 | 49.27±24.27 |
| 6 | 23.32±1.72 | 18.85±1.91 | 28.73±2.42 | 9.88±2.44 | 997.76±3.66 | 5.54±1.48 | 2.13±0.53 | 36.92±11.82 | 59.97±11.74 | 8.54±4.25 | 19.53±6.36 | 5.24±2.74 | 34.27±10.53 |
| 7 | 26.88±2.28 | 23.61±2.32 | 31.02±3.00 | 7.41±2.75 | 997.62±3.39 | 5.18±1.80 | 2.04±0.65 | 50.33±14.63 | 69.66±11.44 | 6.07±4.30 | 15.11±6.51 | 6.53±2.66 | 27.78±11.58 |
| 8 | 27.54±2.93 | 24.04±2.91 | 31.96±3.42 | 7.91±2.21 | 997.29±3.83 | 5.06±1.65 | 1.95±0.67 | 44.74±12.03 | 66.51±9.36 | 7.26±3.86 | 15.55±5.35 | 5.60±2.45 | 25.14±10.09 |
| 9 | 22.32±1.83 | 17.93±2.37 | 27.49±2.27 | 9.56±2.70 | 1003.77±4.73 | 4.14±1.10 | 1.95±0.63 | 38.74±12.20 | 60.07±11.08 | 7.69±3.87 | 14.66±5.18 | 4.66±2.95 | 26.95±12.49 |
| 10 | 15.41±3.89 | 10.67±4.22 | 21.06±4.36 | 10.39±2.76 | 1009.26±4.64 | 4.42±1.37 | 1.98±0.68 | 34.95±10.61 | 59.05±10.92 | 7.48±3.13 | 10.87±3.99 | 3.91±3.04 | 33.64±17.05 |
| 11 | 7.37±4.82 | 3.48±4.97 | 12.12±5.12 | 8.63±2.78 | 1012.63±3.92 | 4.38±1.14 | 2.01±0.76 | 38.26±15.87 | 59.82±14.90 | 5.48±3.41 | 7.24±3.24 | 4.83±3.19 | 42.55±18.87 |
| 12 | 0.22±4.47 | -3.66±4.58 | 4.68±4.65 | 8.33±2.24 | 1015.23±5.48 | 4.33±1.39 | 2.04±0.79 | 34.88±13.91 | 55.20±14.39 | 5.83±3.20 | 6.68±2.80 | 4.15±3.06 | 42.78±18.05 |
| Total | 13.15±10.96 | 8.87±11.03 | 18.25±11.17 | 9.38±2.95 | 1006.24±7.90 | 5.29±1.85 | 2.17±0.76 | 35.14±14.75 | 57.13±14.38 | 7.27±3.91 | 13.43±6.76 | 4.61±3.04 | 41.77±22.50 |

PM10: particulate matter measuring ≤10 μm in diameter
